# Surface Valence State Effect of MoO_2+_ *_x_* on Electrochemical Nitrogen Reduction

**DOI:** 10.1002/advs.202104857

**Published:** 2022-02-20

**Authors:** Jiaqi Wang, Zhou Jiang, Guiming Peng, Eli Hoenig, Gangbin Yan, Mingzhan Wang, Yuanyue Liu, Xiwen Du, Chong Liu

**Affiliations:** ^1^ Pritzker School of Molecular Engineering University of Chicago Chicago IL 60637 USA; ^2^ Institute of New Energy Materials School of Materials Science and Engineering Tianjin University Tianjin 300072 China; ^3^ Department of Mechanical Engineering and Texas Materials Institute The University of Texas at Austin Austin TX 78712 USA; ^4^ Key Laboratory of Materials Modification by Laser Ion and Electron Beams (Dalian University of Technology) Ministry of Education Dalian 116024 China

**Keywords:** ammonia yield, cluster, nitrogen reduction reaction, quantitative isotope labeling, valence effect

## Abstract

The valance of Mo is critical for FeMo cofactor in ambient ammonia synthesis. However, the valence effect of Mo has not been well studied in heterogeneous nanoparticle catalysts for electrochemical nitrogen reduction reaction (NRR) due to the dissolution of Mo as MoO_4_^2−^ in alkaline electrolytes. Here, a MoO_2+_ *_x_* catalyst enriched with surface Mo^6+^ is reported. The Mo^6+^ is stabilized by a native oxide layer to prevent corrosion and its speciation is identified as (MoO_3_)*_n_* clusters. This native layer with Mo^6+^ suppresses the hydrogen evolution significantly and promotes the activation of nitrogen as supported by both experimental characterization and theoretical calculation. The as‐prepared MoO_2+_ *_x_* catalyst shows a high ammonia yield of 3.95 µg mg_cat_^−1^h^−1^ with a high Faradaic efficiency of 22.1% at −0.2 V versus reversible hydrogen electrode, which is much better than the MoO_2_ catalyst with Mo^6+^ etched away. The accuracy of experimental results for NRR is confirmed by various control experiments and quantitative isotope labeling.

## Graphical abstract

Surface Mo^6+^ is designed and stabilized on a corrosion‐resistance MoO_2+_ *_x_* catalyst to enable the study of the Mo valence effect on electrochemical nitrogen reduction. The as‐prepared MoO_2+_ *_x_* shows a significantly higher ammonia yield than that of the MoO_2_ with Mo^6+^ etched away, proving the effectiveness of surface Mo^6+^ in catalyzing nitrogen reduction.

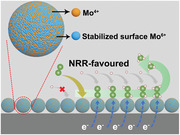

## 1 Introduction

Ammonia (NH_3_) is a potential energy carrier due to its high hydrogen capacity (17.6 wt%) and a crucial commodity feedstock widely used to produce various products such as fertilizers and plastics.^[^ ^1^, ^2^, ^3^ ^]^ To break the extremely strong N≡N triple bond (46.1 kJ mol^−1^), energy‐intensive Haber–Bosch process at high pressures and temperatures (200 atm and 400–450 °C) is used for ammonia production. The Haber–Bosch process consumes ≈1.5% of the global annual energy and releases ≈1.9 tons of CO_2_ per ton of NH_3_ produced yearly.^[^ ^3^, ^4^, ^5^ ^]^ Electrochemical nitrogen reduction reaction (NRR) is an attractive alternative to the Haber–Bosch process.^[^ ^6^ ^]^ It can be carried out under ambient pressure and temperature to significantly reduce the energy consumption and CO_2_ emission, with the utilization of renewable electricity. However, there is lack of catalysts that can induce strong adsorption and efficient activation of N_2_ molecule, which limits the NH_3_ production rate.^[^ ^7^, ^8^ ^]^ Meanwhile, the competitive reaction, hydrogen evolution reaction (HER), is more likely to occur because of the easy activation of proton, thus leading to poor Faradaic efficiency (FE).^[^ ^9^, ^10^, ^11^ ^]^

FeMo cofactor has served as the source of inspiration for ambient nitrogen reduction catalysts for decades due to its highly efficient and selective ammonia conversion capability in aqueous environment.^[^ ^12^, ^13^ ^]^ Molybdenum, therefore, has been investigated in many studies as catalytic centers for nitrogen reduction.^[^ ^13^, ^14^ ^]^ In one synthetic analog, Schrock et al. studied the catalytic reduction of N_2_ to NH_3_ at Mo center of Mo complexes with [HIPTN_3_N]^3−^ ligand (where [HIPTN_3_N]^3−^ is [{3,5‐(2,4,6‐i‐Pr_3_C_6_H_2_)_2_C_6_H_3_NCH_2_CH_2_}_3_N]_3−_).^[^ ^14^ ^]^ In N_2_ conversion to NH_3_, the Mo center was cycled between Mo(III) and Mo (VI). The ability of Mo to adjust its valance is vital to complete the distal pathway to add six electrons and six protons to produce NH_3_. Many efforts, such as doping,^[^ ^13^, ^15^ ^]^ morphological control,^[^ ^16^, ^17^ ^]^ single atom design,^[^ ^18^, ^19^ ^]^ and vacancy creation ^[^ ^20^ ^]^ were made by now to improve the NRR performance of Mo‐based catalysts.

In aqueous heterogeneous electrocatalysis, it is important to investigate whether a solid form of molybdenum can function in a similar mechanism to catalyze the NRR. Due to the strong competition from HER, alkaline electrolytes are preferred to suppress the HER. However, the thermodynamically stable form of molybdenum in alkaline aqueous electrolyte (e.g., pH 13–14) is MoO_4_^2−^ as a soluble ion that is not suitable for heterogeneous catalysis.^[^ ^21^ ^]^ It is known in molybdenum metal corrosion that the solid oxidative forms of molybdenum, mainly Mo(IV)O_2_ in alkaline conditions with co‐existence of Mo(IV)O(OH)_2_ and Mo(VI)O_3_, can occasionally form a passive layer to prevent the dissolution of MoO_4_^2−^.^[^ ^22^, ^23^ ^]^ Therefore, it is possible to investigate molybdenum with different valence states as heterogeneous catalysts in alkaline electrolytes if a self‐limiting oxide layer is formed.

**Figure 1:**
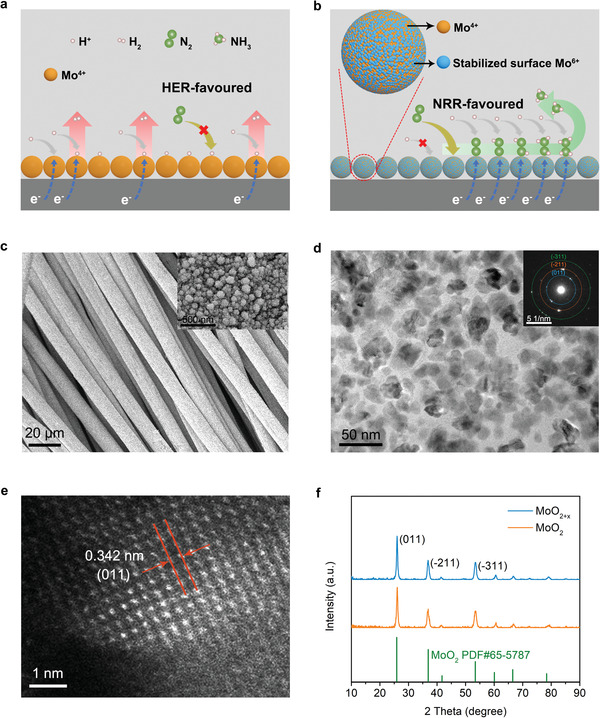
The schematic of NRR and HER on a) pristine MoO_2_ and b) MoO_2+x_ enriched with surface Mo^6+^. c) SEM image of MoO_2+_ *_x_*/CC, the inset is the zoomed‐in SEM image. d) TEM image of MoO_2+_ *_x_*, the inset is the SAED pattern. e) HAADF‐STEM image of MoO_2+_ *_x_*. f) XRD patterns of MoO_2+_ *_x_* and MoO_2_.

In this work, we adopt a hydrothermal method (Figure S1, Supporting Information) to grow MoO_2+_ *_x_*. The direct reduction of Mo(VI)O_4_^2−^, grants the MoO_2_ to be enriched with surface Mo^6+^ (denoted as MoO_2+_ *_x_*). X‐ray photoelectron spectroscopy (XPS), open circuit voltage (OCV), and charge transfer resistance measurements all revealed the presence of surface Mo^6+^. Fourier‐transform infrared spectroscopy (FT‐IR) characterization determined that the existence form of Mo^6+^ is (MoO_3_)*_n_* clusters. The surface Mo^6+^ on MoO_2_ is stable in pH 13 solution while the control Mo(VI)O_3_ dissolves immediately. These surface Mo^6+^ can be etched away to form MoO_2_ in highly concentrated KOH solution (5 m) to allow us to investigate the valance effect of Mo on NRR. In operando voltage, both Mo^6+^ and Mo^4+^ showed reduction to Mo^3+^. While the lack of oxidation peak and after reaction XPS characterization indicates a possible self‐oxidation mechanism allowing Mo^3+^ to convert back to its original Mo^6+^ and Mo^4+^ state, therefore, shuttling between lower and higher valances. When used as an NRR catalyst, the as‐prepared MoO_2+_ *_x_* showed a high NH_3_ yield of 3.95 µg mg_cat._^−1^h^−1^ with a high FE of 22.1% at −0.2 V versus reversible hydrogen electrode (RHE), which is much better than the control MoO_2_ catalyst of 1.06 µg mg_cat_^−1^h^−1^ yield and 9.4% FE, indicating that the remarkable NRR activity originated from the high‐valent Mo^6+^ on the surface. Without surface Mo^6+^, protons can be transferred to the MoO_2_ surface readily, and HER activity is much higher (**Figure** **1**a). With surface Mo^6+^, the HER is suppressed, and nitrogen adsorption is promoted (Figure 1b). Density functional theory (DFT) calculation revealed that with surface attached (MoO_3_)_3_ cluster, the activation barrier for the first hydrogen addition step is lowered. Additionally, both distal and alternative pathways are possible for ammonia synthesis, with the distal path similar to that of the molecular Mo catalyst. It is worth mentioning that the accurate detection of ammonia and the evaluation of catalytic performance remains one of the major challenges of the NRR field. Generally, ammonia production is too low to be firmly attributed to NRR rather than ammonia contamination from air, electrolyte, reduction of other nitrogen species, or catalyst itself.^[^ ^24^, ^25^ ^]^ Strict protocols need to be followed to ensure the measurement accuracy for gaining any fundamental understanding of the catalytic processes.^[^ ^24^ ^]^ Our ammonia yield is assessed by tracking NH_3_ production continuously over 4 h, and we determined the average NH_3_ yield rate by the slope of the yield_NH3_–time plot. This method can reduce test errors significantly compared to the single‐point average method. Moreover, besides all the control experiments, quantitative isotope labeling experiments were performed to track the nitrogen source of yielded ammonia, where purified ^15^N_2_ was used as the feed gas for electrochemical nitrogen reduction. The yield from the quantitative isotope labeling experiment is 3.91 µg mg_cat._^−1^h^−1^ which matches the NH_3_ yield determined by the slope of the yield_NH3_–time plot. This work provided solid evidence pointing to high valence Mo^6+^ being critical for promoting nitrogen reduction

## 2 Results and Discussion

The as‐produced MoO_2+_ *_x_* was thoroughly characterized to determine the material morphology and crystal structure. The scanning electron microscopy (SEM) image shows that the MoO_2+_ *_x_* grows and coats on the carbon cloth (Figure S2, Supporting Information) uniformly with intimate adhesion (Figure 1c). According to the SEM image in Figure S3, Supporting Information and inset in Figure 1c, the sample consists of spherical particles with an average size of 100 nm, formed by agglomeration of smaller particles. The energy‐dispersive spectroscopy mapping demonstrates that Mo and O elements distribute evenly on the carbon cloth (Figure S4, Supporting Information). The transmission electron microscopy (TEM) images of the MoO_2+_ *_x_* in Figure 1d and Figure S5, Supporting Information show small primary particles. The selected area electron diffraction (SAED) pattern shows three distinct rings corresponding to (011), (−211), and (−311), and is indexed along [0−11] zone axis (inset of Figure 1d). Clear lattice fringes could be observed from the aberration‐corrected high‐angle annular dark field scanning TEM (HAADF‐STEM) image in Figure 1e. The lattice spacing of 0.342 nm also corresponds to the (011) plane of MoO_2_.

**Figure 2:**
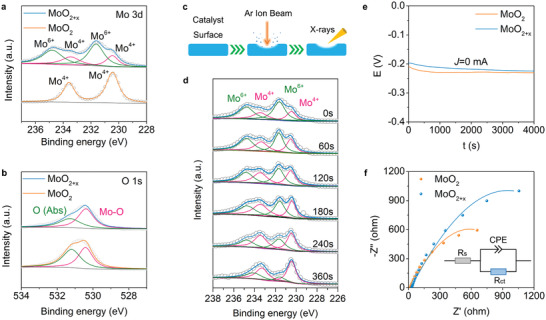
a) Mo 3d XPS spectra of MoO_2+_ *_x_* and MoO_2_. b) O 1s XPS spectra of MoO_2+_ *_x_* and MoO_2_. c) Schematic image of Ar ion depth etching. d) Mo 3d XPS spectra of MoO_2+_ *_x_* recorded after Ar ion depth etching for a series time. e) Open‐circuit voltage (OCV) behavior of MoO_2+_ *_x_* and MoO_2_. f) Electrochemical impedance spectroscopy (EIS) curves of MoO_2+_ *_x_* and MoO_2_ at OCV.

To investigate the effect of Mo valance, an etched sample (denoted as MoO_2_) was prepared by etching MoO_2+_ *_x_* sample in 5 m KOH solution for 24 h to remove Mo (VI). X‐ray diffraction (XRD) patterns reveal that both MoO_2+_ *_x_* and MoO_2_ possess a monoclinic MoO_2_ phase (PDF No. 65‐5787) (Figure 1f). Moreover, the SEM images (Figure S6, Supporting Information) and the TEM images (Figure S7, Supporting Information) of MoO_2_ sample show the unchanged morphology and exposed surface comparing to MoO_2+_ *_x_*. The valance states were confirmed by XPS. As shown in **Figure** **2**a, for MoO_2+_ *_x_*, the peaks at the binding energies of 233.4/230.4 eV are ascribed to Mo^4+^ (3d_3/2_/3d_5/2_), and the other two obviously stronger peaks at 234.7/231.7 eV are assigned to Mo^6+^ (3d_3/2_/3d_5/2_), revealing Mo^6+^ species dominated the surface of the catalyst (65.9%). While for MoO_2_ sample, only Mo 3p_3/2_ and Mo 3p_5/2_ peaks of Mo^4+^ are seen at 233.4 and 230.4 eV, indicating that there is no Mo^6+^ on the surface of MoO_2_ after high concentration KOH etch. Simultaneously, we used inductively coupled plasma mass spectrometry (ICP‐MS) to quantify the mass loss of Mo for sample subjected to the long‐term high concentration KOH etch and calculated that the ratio of Mo^6+^ in sample is 1.83%, which is much lower than the ratio from XPS result, meaning most of Mo^6+^ mainly distribute on the surface of sample rather than the inside. In parallel, the O 1s spectra (Figure 2b) of MoO_2+_ *_x_* and MoO_2_ were split into two peaks at 530.4 and 531.3 eV, corresponding to lattice O (oxygen bonds of Mo—O) and adsorbed O, respectively, consistent with Raman results in Figure S8, Supporting Information. Ar ion depth etching experiment (Figure 2c) was further undertaken to study the composition of MoO_2+_ *_x_* and the etching time was varied to get the signals from different depths of MoO_2+_ *_x_*. Initially, four peaks related to Mo^4+^ and Mo^6+^ could be detected, and the intensity of Mo^6+^ peaks are much higher than those of Mo^4+^, implying the upmost surface of MoO_2+_ *_x_* is mainly composed of Mo^6+^. As the Ar ion bombarding time increases, the ratio of Mo^6+^ declines gradually to a negligible level, while Mo^4+^ becomes dominant in 360 s. These results imply a descending gradient of Mo^6+^ from the surface to the inside, and the inside of MoO_2+_ *_x_* is still composed of Mo^4+^. In comparison with MoO_2_, the Mo^4+/3+^ redox peak of MoO_2+_ *_x_* positively shifted by 90 mV (Figure S9, Supporting Information), revealing a higher Mo valence state than 4+. This observation is consistent with the OCV result in Figure 2e, which shows that MoO_2+_ *_x_* has a higher rest voltage than MoO_2_ because of a higher Mo valence state. Besides, electrochemical impedance spectroscopy measurements at OCV showed that the charge transfer resistance of MoO_2+_ *_x_* was significantly higher than that of MoO_2_ (Figure 2f), suggesting the higher corrosion resistance of MoO_2+_ *_x_* making the surface oxide layer stable.

**Figure 3:**
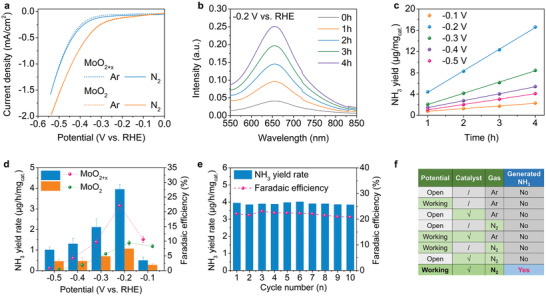
NRR electrochemical performance of MoO_2+_ *_x_*. a) LSV curves of MoO_2+_ *_x_* and MoO_2_ in N_2_‐saturated and Ar‐saturated 0.1 m KOH electrolytes. b) UV−vis spectra of the electrolyte stained with indophenol indicator at different time durations at the potential of −0.2 V versus RHE by using MoO_2+_ *_x_* as catalyst. c) Continuous ammonia yield of MoO_2+_ *_x_* as a function of time at applied potentials from −0.1 to −0.5 V versus RHE. d) NH_3_ yields and FEs of MoO_2+_ *_x_* and MoO_2_ at each given potential. e) Cycling stability of MoO_2+_ *_x_* at −0.2 V versus RHE. f) Table of control experiments to confirm the NH_3_ production over the investigated catalysts.

The electrocatalytic nitrogen reduction was examined in a 0.1 m KOH electrolyte using an H‐shape electrolysis cell wherein the two compartments of the cell were separated by a proton exchange membrane, as illustrated in Figure S10, Supporting Information. The electrodes are composed of Ag/AgCl as the reference electrode, a graphite rod as the counter electrode, and a carbon cloth loaded with the MoO_2+_ *_x_* as the working electrode. Before the test, the N_2_ gas was bubbled through a 1 m H_2_SO_4_ aqueous solution (to eliminate the possible NH_3_ impurity) and a 2 m KOH solution (to eliminate the possible NO*_x_* impurity) before being fed into the electrolyte. All the potentials were converted to the RHE. As shown in **Figure** **3**a, linear sweep voltammetry (LSV) curves of MoO_2+_ *_x_* were first collected in both N_2_‐saturated and Ar‐saturated 0.1 m KOH, respectively. The current density of LSV curves in N_2_‐saturated electrolyte is higher than that in Ar‐saturated electrolyte, indicating that the electrochemical reduction of N_2_ has occurred. It is worth mentioning that the HER onsite of MoO_2_ is much earlier than MoO_2+_ *_x_*, indicating a stronger HER competition in the MoO_2_ case. These results show that the surface Mo^6+^ can help suppress the HER because of the higher charge transfer resistance (Figure 2f).

The ammonia production is measured by the indophenol blue method. The concentration of NH_4_^+^ in the electrolyte is calibrated by a standard plot, which shows a highly linear relationship between the absorbance and concentration (Figure S11, Supporting Information). Because of the low NRR ammonia production rate and potential environmental contaminations, it strongly calls for a reliable method to measure the low NRR ammonia production rate to report a solid result.^[^ ^18^, ^19^ ^]^ To avoid the poor reliability issue of the NH_3_ yield determination method by using single point ammonia concentration, in this work, we tracked the continuous ammonia generation over a long‐time range, a method developed in our previous study.^[^ ^11^ ^]^ When using MoO_2+_ *_x_* as the catalyst, the UV–vis results show that the concentration of NH_4_^+^ in the electrolyte increases as time progresses over 4 h for all applied potentials from −0.1 to −0.5 V versus RHE (Figure 3b; Figure S12, Table S1, Supporting Information). Figure 3c shows that the NH_3_ yield increases linearly over time, providing a more accurate way to determine the NH_3_ yield rate according to the slope of yield_NH3_–time plot. This rules out the excess NH_3_ contribution from the background and initial error. At the same time, the total yield at −0.2 V for MoO_2+_ *_x_* over 8 h exceeds 10 times the initial background ammonia concentration confirming that the ammonia production is from NRR (Figure S13, Supporting Information). Figure 3d presents the NH_3_ yield and FE of MoO_2+_ *_x_* and MoO_2_ at various potentials. The highest ammonia yield rate of 3.95 µg mg_cat_^−1^h^−1^ and FE of 22.1% for NRR on MoO_2+_ *_x_* was observed at −0.2 V versus RHE (Figure 3d), which was much higher than that of MoO_2_ of 1.06 µg mg_cat_^−1^h^−1^ yield and 9.4% FE (Figure S14, Supporting Information). We also varied the mass loading of MoO_2+_ *_x_* catalyst from 0.1 to 1.3 mg cm^−2^ (Figure S15, Supporting Information) and tested their NRR performance. As shown in Figure S16 and Table S2, Supporting Information, the highest ammonia yield rate and FE were achieved under the mass loading of 0.9 mg cm^−2^. Moreover, Nessler's reagent detection method was applied to confirm the performance. According to the calibration curves shown in Figure S17, Supporting Information and experimental results shown in Figure S18, Supporting Information, the yield is almost the same with that determined by indophenol blue method.^[^ ^26^ ^]^ Besides, the ammonia yield rate and FE of MoO_2+_ *_x_* were unchanged during ten cycling tests (Figure 3e), and the morphology and the valence state did not change after the long‐term stability test (Figure S19, Supporting Information). Furthermore, no byproduct N_2_H_4_ was detected after the electrochemical reduction (Figure S20, Supporting Information), demonstrating that the MoO_2+_ *_x_* has an excellent selectivity toward NRR. To further verify that the ammonia was produced by reducing nitrogen rather than from the reactants, instrument, air, or electrolyte, we conducted a series of control experiments where only one parameter was varied each time, and the other conditions were kept unchanged (Figure 3f; Figures S21,S22, Supporting Information). The results display that there were no ammonia production without one of the necessary conditions, including catalyst, applied potential, and N_2_, illustrating that ammonia produced in the electrolyte comes from the electrochemical reduction of N_2_ rather than any other contamination.

**Figure 4:**
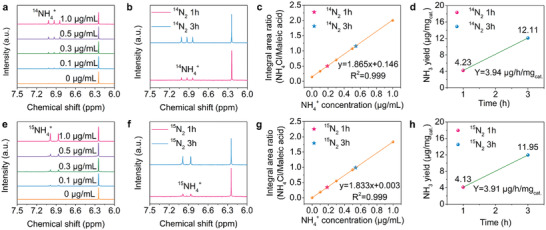
^1^H NMR spectra of standard ^14^NH_4_Cl a) with different concentrations of ^14^NH_4_^+^ and ^15^NH_4_Cl and e) with different concentrations of ^15^NH_4_^+^ using maleic acid as an internal standard. ^1^H NMR spectra for electrolytes after NRR tests at reaction duration of 1 and 3 h when feeding with b) ^14^N_2_ and f) ^15^N_2_ at −0.2 V versus RHE. Corresponding standard curve for standard c) ^14^NH_4_Cl and g) ^15^NH_4_Cl versus peak area ratio. The orange balls represent the values from the standard NH_4_Cl samples, and the pink star and blue star show the position of the NMR‐measured value for electrolytes after NRR tests at reaction duration of 1 and 3 h, respectively. Ammonia yield of MoO_2+_ *_x_* after reaction duration of 1 and 3 h at −0.2 V versus RHE when feeding with d) ^14^N_2_ and h) ^15^N_2_.

Furthermore, quantitative isotopic labeling experiments were performed to analyze the concentration of ^15^N‐ and ^14^N‐labeled NH_4_^+^ by ^1^H NMR spectrum (**Figure** **4**a,e), and the corresponding concentration‐signal integration results showed a good linear relationship (Figure 4c,g). Figure 4b,f shows that the concentration of both ^15^NH_4_^+^ and ^14^NH_4_^+^ in the electrolyte increase as time progressed and the concentration of ^15^NH_4_^+^ was in quantitative agreement with the concentration of ^14^NH_4_^+^ produced under the equivalent conditions. Moreover, the yield measured with the calculated concentration of ^15^NH_4_^+^ is 3.91 µg mg_cat_^−1^h^−1,^ and the yield measured with the calculated concentration of ^14^NH_4_^+^ is 3.94 µg mg_cat_^−1^h^−1^, which are consistent with the result of the UV–vis (Figure 4d,h), suggesting reliable experimental results.

MoO_2+_ *_x_* catalyst exhibits overwhelming superiority in producing ammonia comparing to MoO_2_ catalyst, which points to the presence of surface Mo^6+^ being critical. Cyclic voltammetry (CV) was conducted in inert solvent acetonitrile and inert Ar atmosphere. The CV curves (Figure S9, Supporting Information) showed reduction peaks at −0.30 and −0.39 V for MoO_2+_ *_x_* and MoO_2_. Due to the inert environment, we assign these peaks to the reduction to Mo (III). However, the oxidation part of the CV did not show a comparable peak. XPS spectra of post‐NRR MoO_2+_ *_x_* sample (Figure S19b, Supporting Information) were characterized. The surface valance states of Mo are comparable to that before reaction with similar Mo^6+^ to Mo^4+^ ratio. These results show the possibility that the in operando the Mo^6+^ was reduced to Mo^3+^ and the Mo^3+^ can experience auto‐oxidation to convert back to Mo^6+^, therefore shuttling between different valances as that in FeMo cofactor or its analog.^[^ ^14^ ^]^

To further prove the effect of Mo^6+^, we synthesized another MoO_2+_ *_x_* sample as negative control by increasing the mass of CTAB during synthesis and found the morphology and phase keep unchanged, but the ratio of Mo^6+^ decreased from 65.9% to 38.2% significantly than before (Figure S23a–c, Supporting Information). The ammonia yield rate of 2.74 µg mg_cat_^−1^h^−1^ and FE of 15.7% for NRR are also lower than those of MoO_2+_ *_x_* (65.9% Mo^6+^), further proving the pivotal role and the optimal content of Mo^6+^ in MoO_2+_ *_x_* for NRR (Figures S23d,S24, Supporting Information).

**Figure 5:**
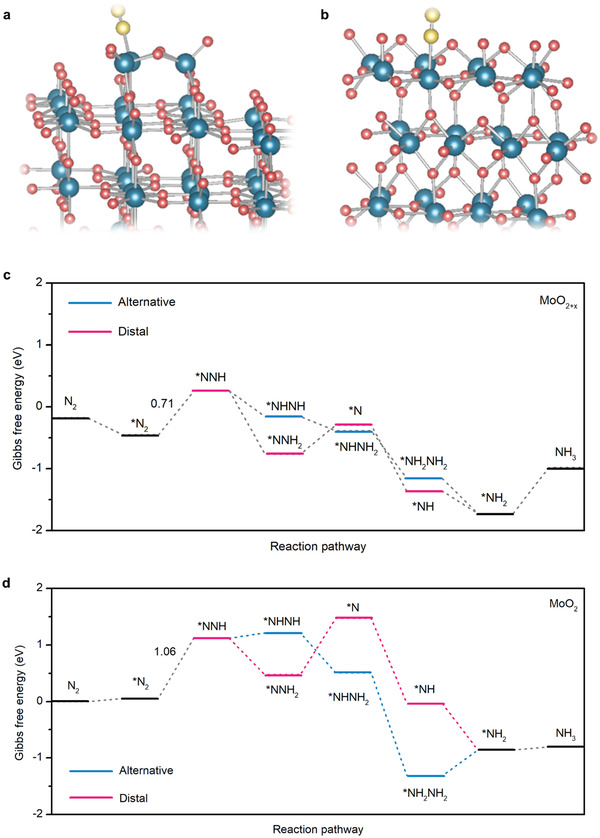
Atomic models of a) MoO_2+_ *_x_* and b) MoO_2_ with a dinitrogen molecule adsorbed for DFT simulation. Blue, red, and yellow atoms represent Mo, O, and N atoms, respectively. Calculated Gibbs free energies of the NRR on c) MoO_2+_ *_x_* and d) MoO_2_ along the alternative and distal pathways.

To gain insight into the nature of the high NRR activity of MoO_2+_ *_x_* enriched with Mo^6+^, systematical DFT calculations were implemented. We first exploited FT‐IR to characterize the speciation of surface Mo^6+^. As shown in Figure S25, Supporting Information, MoO_2+_ *_x_* has the obvious absorption peak of (MoO_3_)*_n_* cluster but MoO_2_ does not.^[^ ^27^ ^]^ Then we constructed a (MoO_3_)_3_ cluster‐MoO_2_ (011) surface to simulate the MoO_2+_ *_x_* (**Figure** **5**a). Meanwhile, normal MoO_2_ (011) surface model was built to represent MoO_2_ (Figure 5b). The NRR process on the catalyst was proposed as hydrogenation of the adsorbed N_2_ molecule by the addition of H atoms one by one. Figure S26, Supporting Information shows that the NRR can proceed through two pathways: the alternative pathway and distal pathway. In the alternative pathway, the two nitrogen atoms are hydrogenated one by one, whereas in the distal pathway one N is fully hydrogenated before the second N. For both pathways, the first hydrogenation (*NN → *NNH) process is the rate‐limiting step, determining the NRR performance (Figure 5b). Introducing Mo^6+^ could increase the adsorption of dinitrogen molecular significantly and reduce the energy barriers for the first hydrogen addition step (*N_2_ to *NNH: from 1.06 to 0.71 eV), compared to the pure MoO_2_. The free energy change for this step is comparable to those in literature.^[^ ^28^, ^29^, ^30^ ^]^ Note that the effects of dynamic surface charge and explictic solvation were not considered here due to the computational cost; these factors could further lower the formation energy of *NNH.^[^ ^31^, ^32^, ^33^, ^34^, ^35^, ^36^ ^]^ This finding is consistent with the experimental result and explains why the MoO_2+_ *_x_* catalyst has higher NRR performance.

## 3 Conclusion

In summary, we prepared MoO_2+_ *_x_* enriched with surface Mo^6+^, which showed a high NH_3_ yield of 3.95 µg mg_cat._^−1^h^−1^ with a high FE of 22.1% at −0.2 V versus RHE. Both experimental characterization and DFT calculations revealed that the remarkably enhanced NRR activity originated from the high‐valent Mo^6+^ on the surface. Moreover, various control experiments and quantitative isotope labeling experiments were performed to ensure the accuracy of experimental results for electrochemical nitrogen reduction. This work demonstrates that creating unique valence states is an efficient approach to design excellent electrocatalysts for nitrogen reductyield_NH3_‐timeion.

## 4 Experimental Section

The experiments and methods are described in the Supporting Information.

## Conflict of Interest

The authors declare no conflict of interest.

## Author Contributions

C.L. and J.W. designed the project. J.W. performed the experiments. G.P. helped with UV–vis and NRR experiments. E.H. helped with TEM and SEM characterization. G.Y. helped with ICP‐MS and XRD characterization. M.W. helped with Raman characterization. J.W. and C.L. performed the experimental data analysis. Z.J. and Y.L. performed the theoretical calculation. J.W. and C.L. wrote the paper. All authors discussed the results and commented on the manuscript. C.L., X.D., and Y.L. supervised the project.

## Supporting information

Supporting Information

## Data Availability

The data that support the findings of this study are available from the corresponding author upon reasonable request.
