# Supplementary material for: Surface Valence State Effect of MoO2+ x on Electrochemical Nitrogen Reduction
Source: Adv Sci (Weinh). 2022 Feb 20;9(12):2104857. doi: 10.1002/advs.202104857 (PMC9036006; doi:10.1002/advs.202104857)
Supplement: Supplementary file 1 — Supporting Information [file ADVS-9-2104857-s001.pdf]

ADVANCED SCIENCE
Open Access

Supporting Information

for *Adv. Sci.*, DOI 10.1002/advs.202104857

Surface Valence State Effect of MoO_{2+x} on Electrochemical Nitrogen Reduction

Jiaqi Wang, Zhou Jiang, Guiming Peng, Eli Hoenig, Gangbin Yan, Mingzhan Wang, Yuanyue Liu, Xiwen Du* and Chong Liu**

Supporting Information

Surface Valence State Effect of MoO_{2+x} on Electrochemical Nitrogen Reduction

Jiaqi Wang, Zhou Jiang, Guiming Peng, Eli Hoenig, Gangbin Yan, Mingzhan Wang, Yuanyue Liu, Xiwen Du*, Chong Liu**

J.Q. Wang, G.M. Peng, E. Hoenig, G.B. Yan, M.Z. Wang, Prof. C. Liu

Pritzker School of Molecular Engineering, University of Chicago,

Chicago, Illinois 60637, United States

E-mail: chongliu@uchicago.edu

J.Q. Wang, Prof. X.W. Du

Institute of New Energy Materials, School of Materials Science and Engineering, Tianjin University,

Tianjin 300072, China

E-mail: xwdu@tju.edu.cn

Z. Jiang, Prof. Y.Y. Liu

Texas Materials Institute, The University of Texas at Austin,

Austin, Texas 78712, United States

E-mail: yuanyue.liu@austin.utexas.edu

Z. Jiang

Key Laboratory of Materials Modification by Laser, Ion and Electron Beams, Dalian University of Technology,

Dalian 116024, China

Experimental section

Preparation of MoO_{2+x}/CC. The MoO_{2+x} was synthesized by a simple hydrothermal method. 0.8 g of ammonium molybdate tetrahydrate (Chem-Impex, 99.8%) was dissolved in 50 mL of distilled water under stirring. Then 1.5 mL HNO_3 (Fisher-Chemical, 67%) was added dropwise to the solution. Additional 5 mL of CTAB (0.08 g) (Bioworld, 98%) ethanol solution was added to the above solution. The solution was next transferred in a Teflon-lined stain-less autoclave in which a 1 cm × 3 cm carbon cloth (CC) was placed in advance. The autoclave was placed in a furnace at 180 °C for 24 h, and naturally cooled down to room temperature. Finally, the carbon cloth was washed thoroughly with distilled water and ethanol several times to eliminate possible remnants and dried in a vacuum at 80 °C for 10 h to get the final MoO_{2+x}/CC. MoO_{2+x} loading was measured by the mass difference before and after synthesis.

Preparation of MoO_2/CC. The as-prepared MoO_{2+x}/CC was immersed in 5M KOH (Sigma-Aldrich, 98%) solution at room temperature for 24h. Then, the carbon cloth was washed thoroughly with distilled water and ethanol several times, and dried in a vacuum at 80 °C for 10 h to get the final MoO_2/CC.

Preparation of MoO_{2+x}/CC (less Mo^{6+}). MoO_{2+x}/CC (less Mo^{6+}) was synthesized following the same procedures as previous MoO_{2+x}/CC except the mass of CTAB was 0.12 g.

Material characterizations. Scanning electron microscopy (SEM, Zeiss Merlin) was performed at the accelerating voltage of 10 kV. Transmission electron microscopy (TEM) images were taken on a FEI Tecnai T12 microscope with accelerating voltage of 120 kV. STEM images were acquired using JEOL ARM 200F equipped with a cold field emission source. HAADF images were acquired at 200 kV with a less than 0.8 Å spatial resolution. X-ray diffraction patterns were obtained on Bruker D8 Powder X-ray diffractometer. X-ray photoelectron spectroscopy measurements were carried out on Kratos AXIS Nova based on a monochromatic Al Kα X-ray source. Raman spectroscopy was collected on a Horiba LabRAM Raman microscopy at the excitation of 633 nm. UV-vis spectra were collected on a Shimadzu UV-3600 Plus UV-Vis-NIR spectrophotometer. The sample compositions were diluted in a 3% HNO_3 matrix and measured using either Thermo iCAP Q ICP-MS or Thermo iCAP RQ ICP-MS.

Electrochemical experiments. The nitrogen reduction reaction (NRR) experiments were carried out using a three-electrode Nafion membrane-separated H-cell on a BioLogic VMP3 potentiostat system. Note that the Nafion membrane was pretreated in 5% H_2O_2 aqueous solution at 80 °C and ultrapure water at 80 °C successively before use. It was kept in each solution for one hour. Ag/AgCl (saturated KCl) electrode is used as reference electrode. Counter electrode is graphite rod. The electrolyte is 0.1M KOH aqueous solution. To remove the trace NH_3 impurity, N_2 gas (99.99%) was bubbled through 1M H_2SO_4 aqueous solution and 2M KOH solution before fed into the electrolyte. The electrolyte is saturated with N_2 for 30 minutes ahead of NRR experiments. During NRR experiment, a constant N_2 flow was fed into the electrolyte at the cathode half-cell. All potentials were converted into values versus reversible hydrogen electrode (RHE) following the Nernst equation.

Determination of ammonia concentration via indophenol blue colorimetry. The concentration of ammonia in electrolyte was determined using the indophenol blue method. Specifically, 1 mL of the electrolyte was added into 1 mL solution which was composed of 1 M NaOH and 5 wt% salicylic acid and 5 wt% sodium citrate. Then 0.5 mL of 0.05 M NaClO aqueous solution was added, followed by addition of 0.1 mL of sodium nitroferricyanide (1 wt%) aqueous solution. After settling for 1 hour, the UV-vis spectra were collected. The NH_3 production was indicated by the formation of indophenol blue, which was determined by the absorbance at 655 nm, with its concentration calibrated by a standard plot using a serial of concentration-known ammonium chloride (Alfa-Aesar, 99.5%) solutions.

Determination of ammonia concentration via Nessler colorimetry. Different concentrations of ammonia-nitrogen solutions were prepared in a vial and topped off with 1 mL using 0.1 M KOH solution. Then, 0.1 mL Nessler's reagent (Macklin, 99.9%) and 0.1 mL 0.2 M potassium sodium tartrate (Sigma-Aldrich, 99.9%) aqueous solution were added into the different ammonia-nitrogen solutions. Finally, the UV-vis spectrophotometry of the different solutions was determined at 420 nm after resting for 20 min.

NMR determination of ammonia. The NH_3 concentration was also quantitatively determined by ^{1}H nuclear magnetic resonance (Agilent 400-MHz system) with using DMSO-d_6 (Millipore-Corporation, 99.8%) as a solvent and maleic acid ($C_4H_4O_4$, Sigma-Aldrich, 99.5%) as the internal standard. The calibration curve was made as follows. Firstly, a series of

ammonium chloride solutions with known concentration were prepared in 0.1 M KOH; secondly, 0.225 mL of the standard solution and 0.05 mL of DMSO-d_6 was mixed with 0.225 mL 0.2 M HCl (1 mM $C_4H_4O_4$); thirdly, the mixture was tested by NMR spectrometer at room temperature; finally, the calibration curve was achieved using the peak area ratio between NH_4^+ and $C_4H_4O_4$ because the NH_4^+ concentration and the area ratio are positively correlated. For testing the produced NH_4^+ from NRR, the processes of testing produced NH_4^+ are the same to that for making the calibration curve except the ammonium chloride solution was replaced by the obtained electrolyte after the test. Then, the amount of produced NH_4^+ can be calculated from the peak area using the calibration curve.

$^{15}N_2$ Isotope Labeling experiment. The $^{15}N_2$ isotope labeling experiments were carried out using $^{15}N_2$ gas as reactant for electrochemical NRR. $^{15}N_2$ gas was first purged through 1M H_2SO_4 solution and 2M KOH solution to eliminate the potential contaminants before the electrocatalytic tests. After 1 h and 3h electrochemical NRR in 0.1 M KOH solution under the potential of −0.2 V versus RHE, 1 mL post electrolyte solutions were mixed with HCl, maleic acid and DMSO-d_6 solutions and used for ^1H NMR measurement. For quantitative comparison, the same operation was performed for $^{14}N_2$ as feed gas.

Determination of hydrazine concentration. The hydrazine was detected using the method of Watt and Chrisp as following. Firstly, a color reagent was prepared by mixing 3 g of para-(dimethylamino) benzaldehyde and 15 mL of 12 M HCl aqueous solution into 150 mL of absolute ethanol. Then 1 mL of the above solution was mixed with 1 mL of the electrolyte in our study. After settling for 10 minutes, the mixture was detected by the UV-vis spectroscopy at the wavelength of 455 nm.

Computational Method. The DFT calculations were performed within the generalized gradient approximation (GGA) of Perdew-Burke-Ernzerhof (PBE) functional in Vienna Ab initio Simulation Package (VASP).[1-3] Core electron states were described by the projector augmented-wave method (PAW) with a kinetic energy cutoff of 500 eV for the planewave expansion.[4,5] The convergence criterion was set to 0.02 eV/Å per atom for force and 10^{-5} eV for energy, respectively. The Brillouin zone was sampled using a k-points grid of Monkhorst-Pack with a uniform spacing of 0.025 $Å^{-1}$. According to XRD, MoO_2 (011) surface with oxygen atom having both one and two coordinates was chosen to investigate the steps of the N_2

reduction reaction (NRR), the surface model was constructed with a 2×1×1 supercell containing 3 atom-layer slab, and a 20 Å vacuum along the z direction. NRR involves six net coupled proton and electron transfer steps ($N_2 + 6H^+ + 6e \rightarrow 2NH_3$). The Gibbs free energy change of NRR is calculated by using the standard hydrogen electrode (SHE) model proposed by Nørskov et al.[6-8] The reaction free energies of the NRR steps were calculated as: $G = E_{DFT} + E_{ZPE} - T\Delta S$, where E_{DFT} is the DFT calculated energy, E_{ZPE} and $T\Delta S$ are obtained by DFT vibration frequency calculations. To account for the influence of an applied electric potential on the electrode reaction, the free energy of each step was scaled by -neU, where n is the number of electrons in the reaction and U is the applied bias.

1. Kresse, G.; Furthmüller, J., Efficiency of ab-initio total energy calculations for metals and semiconductors using a plane-wave basis set. *Computational Materials Science* **1996**, 6 (1), 15-50.
2. Kresse, G.; Furthmüller, J., Efficient iterative schemes for ab initio total-energy calculations using a plane-wave basis set. *Physical Review B* **1996**, 54 (16), 11169-11186.
3. Perdew, J. P.; Burke, K.; Ernzerhof, M., Generalized Gradient Approximation Made Simple. *Physical Review Letters* **1996**, 77 (18), 3865-3868.
4. Blöchl, P. E., Projector augmented-wave method. *Physical Review B* **1994**, 50 (24), 17953-17979.
5. Kresse, G.; Joubert, D., From ultrasoft pseudopotentials to the projector augmented-wave method. *Physical Review B* 1999, 59 (3), 1758-1775.
6. Nørskov, J. K.; Rossmeisl, J.; Logadottir, A.; Lindqvist, L.; Kitchin, J. R.; Bligaard, T.; Jónsson, H., Origin of the Overpotential for Oxygen Reduction at a Fuel-Cell Cathode. *The Journal of Physical Chemistry B* **2004**, 108 (46), 17886-17892.
7. Rossmeisl, J.; Logadottir, A.; Nørskov, J. K., Electrolysis of water on (oxidized) metal surfaces. *Chemical Physics* **2005**, 319 (1), 178-184.
8. Peterson, A. A.; Abild-Pedersen, F.; Studt, F.; Rossmeisl, J.; Nørskov, J. K., How copper catalyzes the electroreduction of carbon dioxide into hydrocarbon fuels. *Energy & Environmental Science* **2010**, 3 (9), 1311-1315.

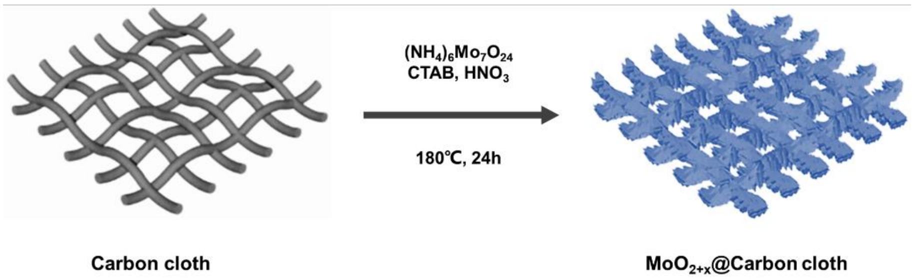

Figure S1. Schematic diagram on the synthesis of MoO_{2+x}/CC by hydrothermal method.

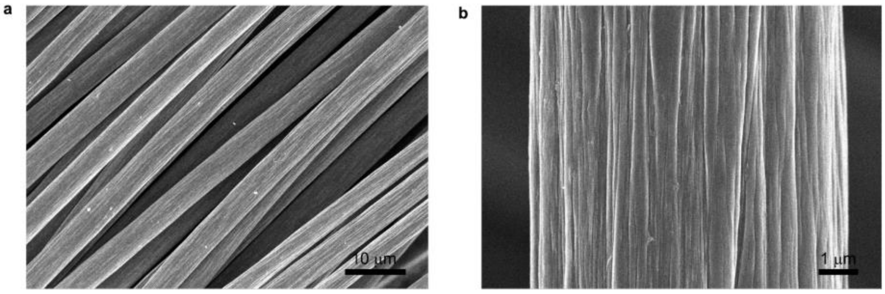

Figure S2. SEM image (a) and zoomed-in SEM image (b) of bare carbon cloth.

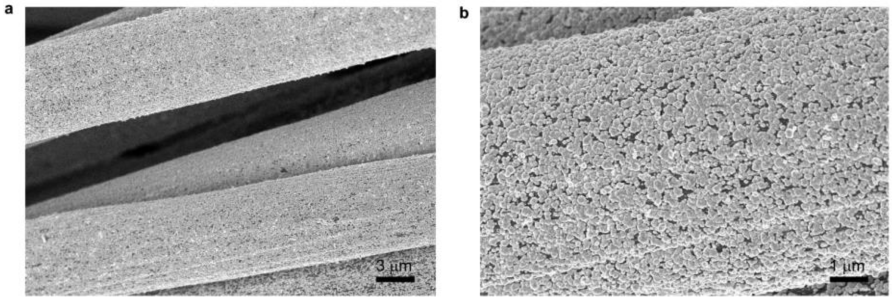

Figure S3. SEM images of MoO_{2+x}/CC on lower resolution (a) and higher resolution (b). The mass loading of MoO_{2+x} is 0.9 mg/cm^2.

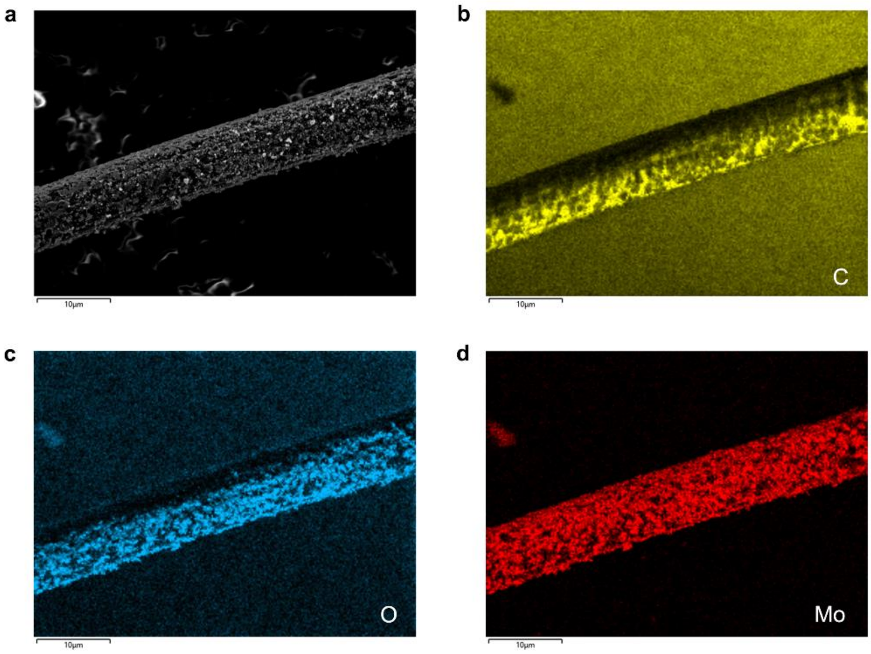

Figure S4. Scanning SEM image (a) of MoO_{2+x}/CC and EDX elemental mapping of C (b), O (c), and Mo (d).

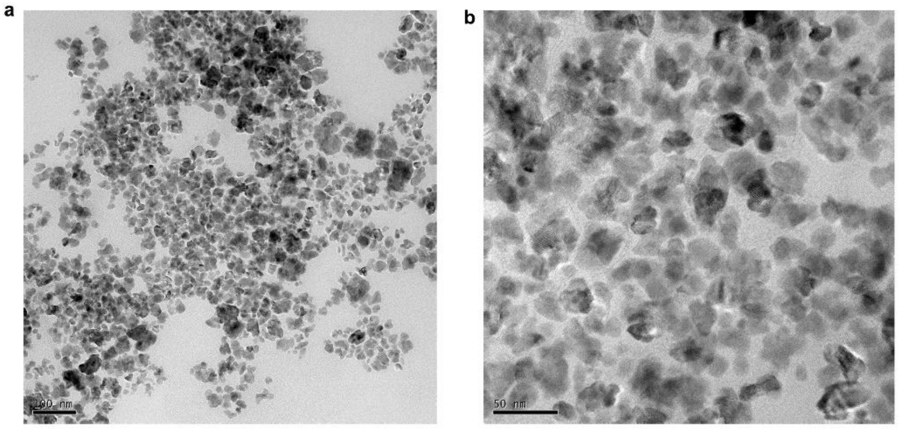

Figure S5. TEM images of MoO_{2+x}.

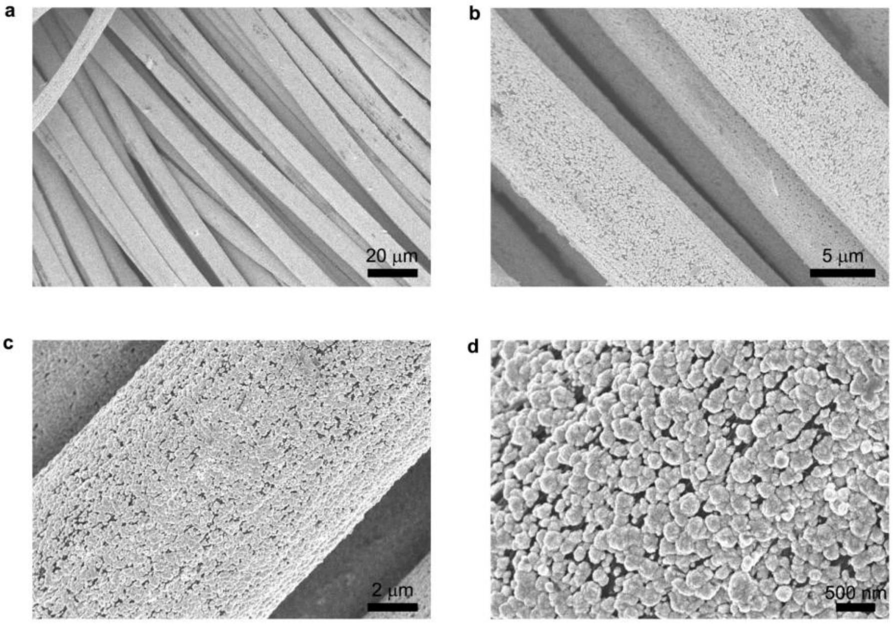

Figure S6. (a-d) SEM images of MoO_2 on higher and higher resolution.

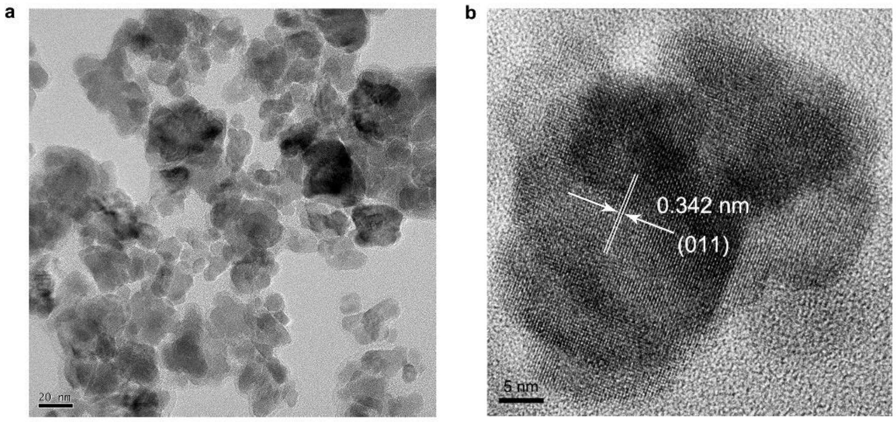

Figure S7. TEM image (a) and HRTEM (b) image of MoO_2.

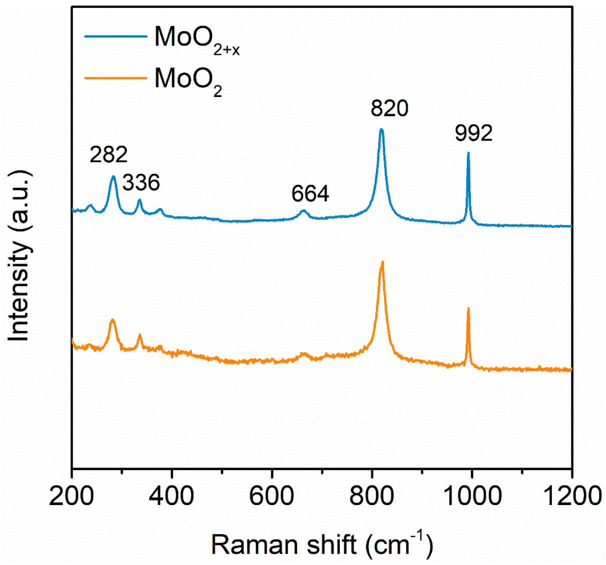

Figure S8. Raman spectra of MoO_{2+x} and MoO_2.

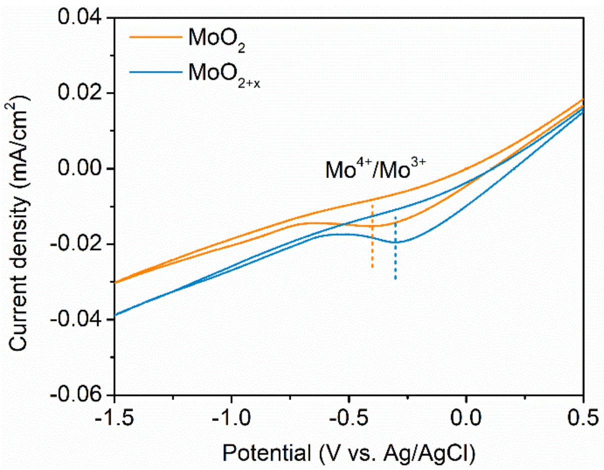

Figure S9. Cyclic voltammetry (CV) curves in Ar-saturated acetonitrile for MoO_{2+x} and MoO_2.

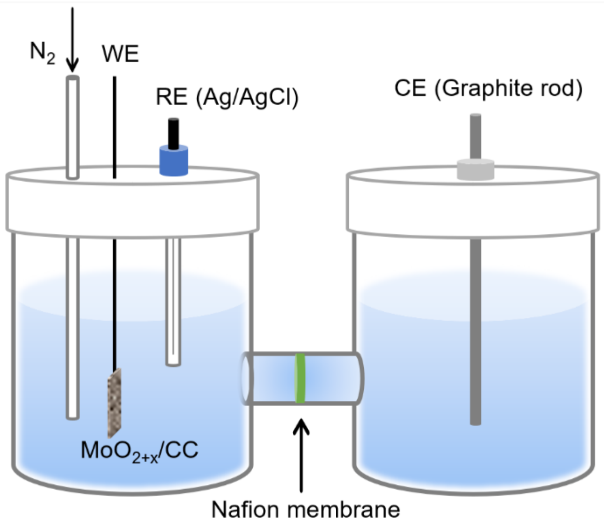

Figure S10. Schematic graph to illustrate the electrocatalytic setup for the NRR test.

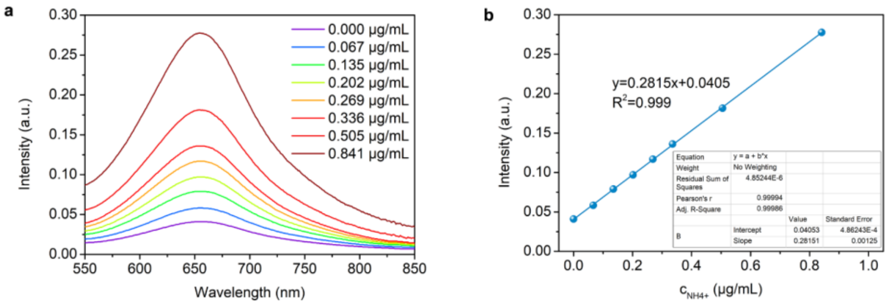

Figure S11. (a) UV-vis spectra at various ammonia concentrations after being incubated for 1 h. (b) The corresponding calibration curve.

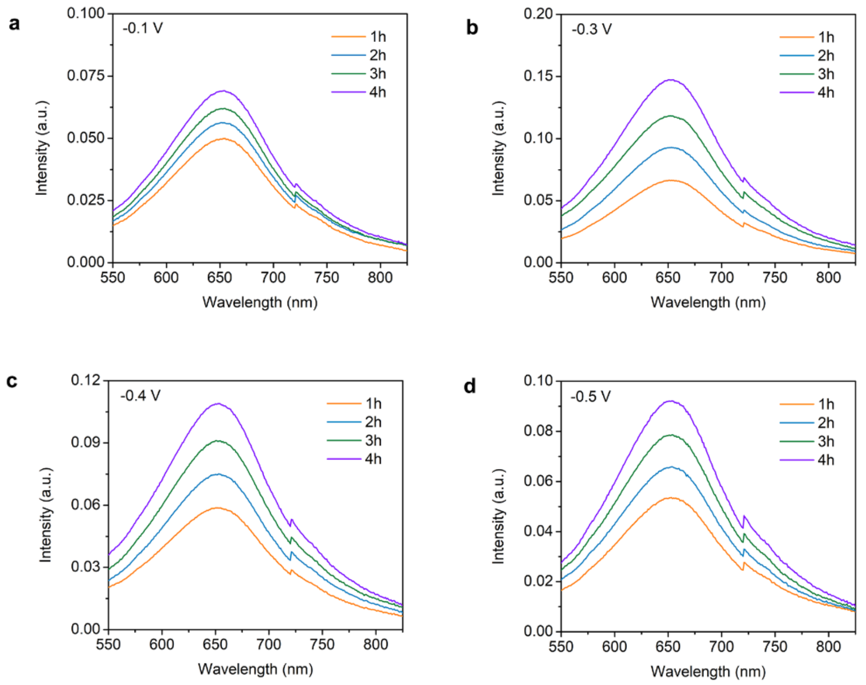

Figure S12. UV-vis spectra of the electrolyte stained with indophenol indicator at different time durations at the potential of −0.1 V (a), -0.3 V (b), -0.4 V (c) and -0.5 V (d) versus RHE by using MoO_{2+x} as catalyst.

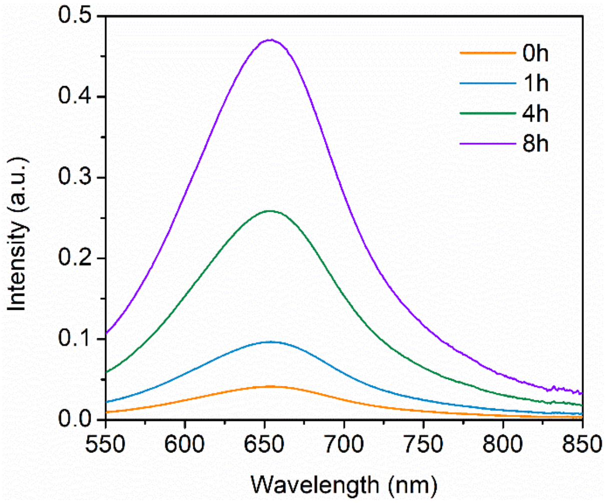

Figure S13. UV-vis spectra of the electrolyte stained with indophenol indicator at −0.1 V over 8h.

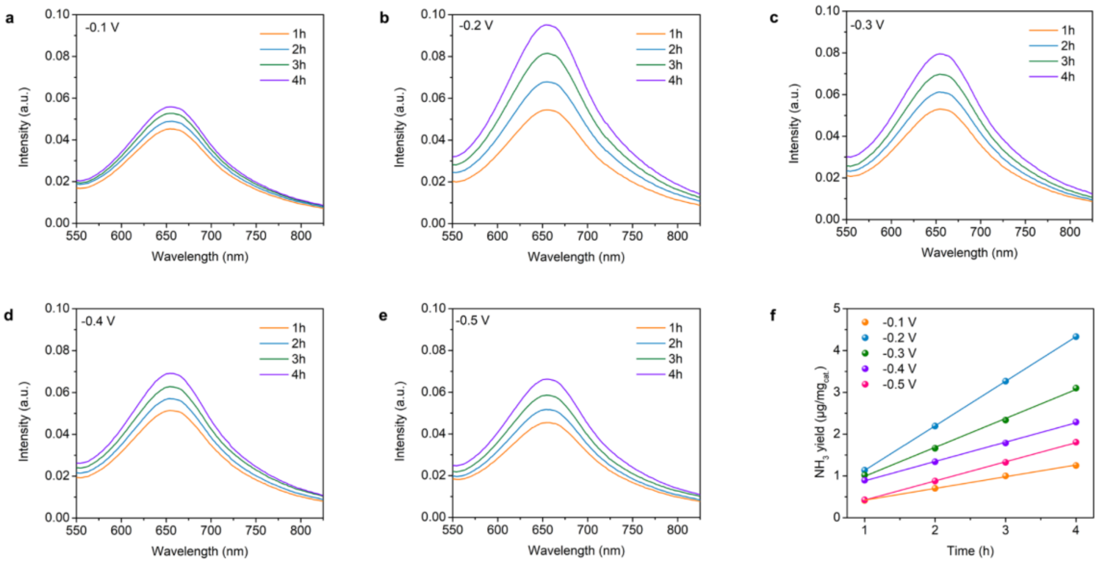

Figure S14. UV-vis spectra of the electrolyte stained with indophenol indicator at different time durations at the potential of −0.1 V (a), -0.2 V (b), -0.3 V (c), -0.4 V (d) and -0.5 V (e) versus RHE by using MoO_2 as catalyst. (f) Continuous ammonia yield of MoO_2 as a function of time at applied potentials from −0.1 V to −0.5 V vs RHE.

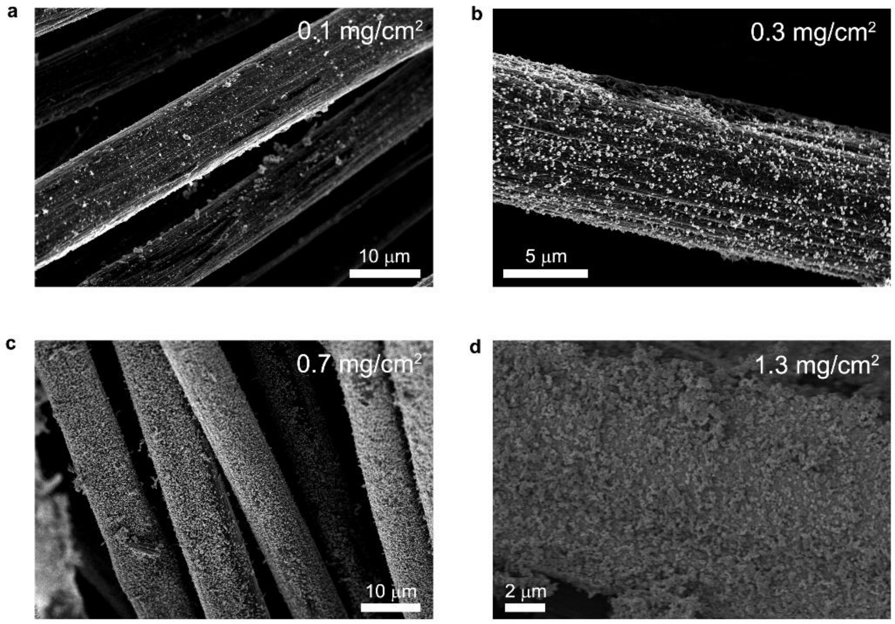

Figure S15. SEM images of MoO_{2+x}/CC with various mass loading of 0.1 mg/cm^2 (a), 0.3 mg/cm^2 (b), 0.7 mg/cm^2 (c) and 1.3 mg/cm^2 (d).

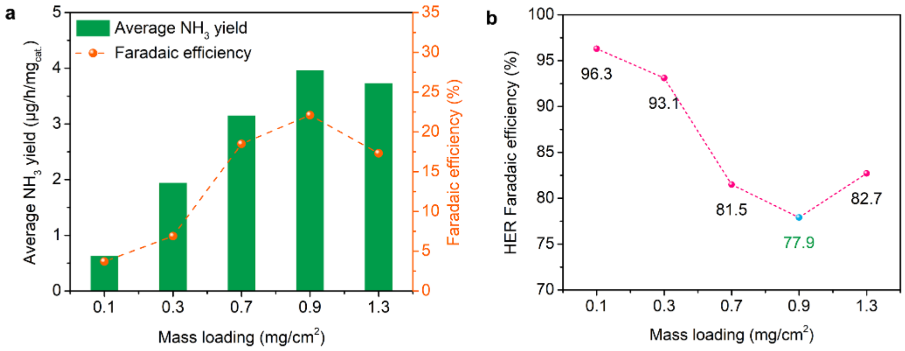

Figure S16. (a) NH_3 yields and FEs of MoO_{2+x} with various mass loading at the potential of -0.2 V vs. RHE. (b) The corresponding HER FEs under various mass loading.

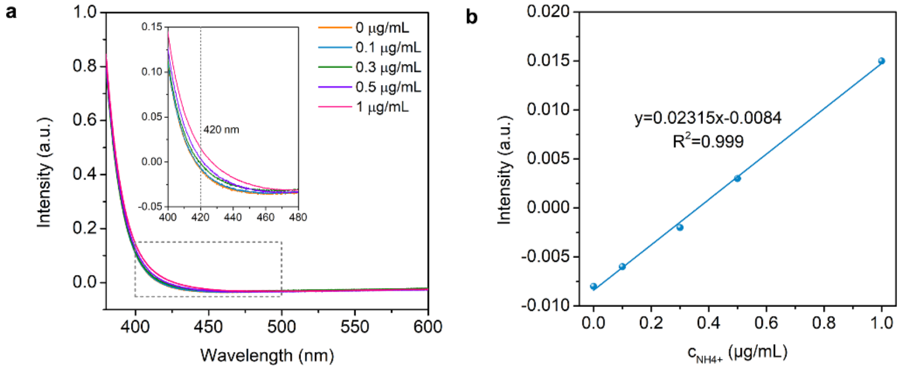

Figure S17. (a) UV-vis spectra at various ammonia concentrations for the colorimetric NH_3 assay using Nessler's reagent. (b) The corresponding calibration curve.

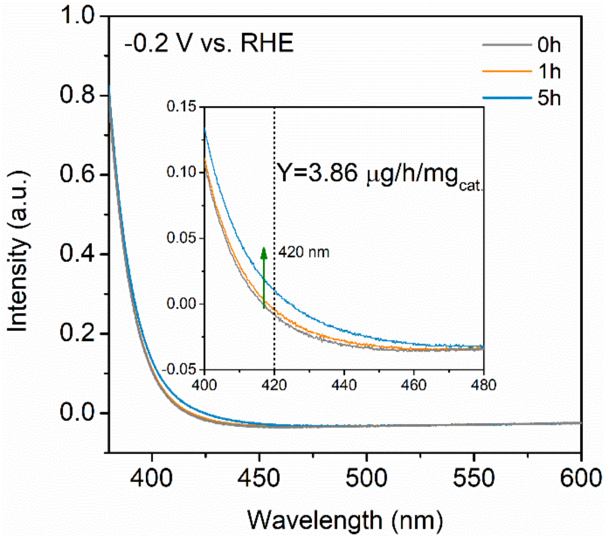

Figure S18. UV-vis spectra of the electrolyte colored with Nessler indicator at -0.2 V over 5h.

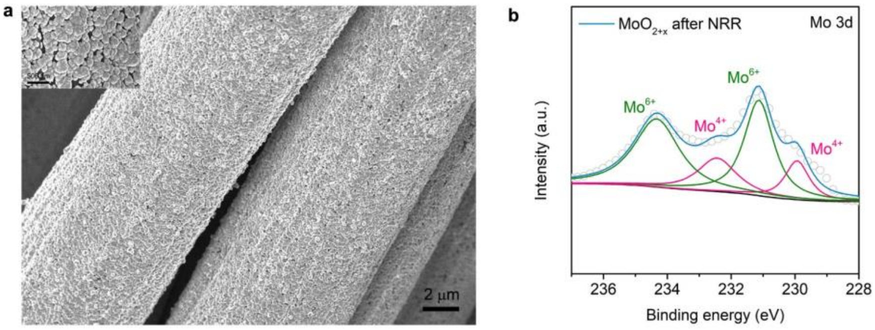

Figure S19. (a) SEM image of MoO_{2+x}/CC after NRR test, the inset is the zoomed-in SEM image. (b) Mo 3d XPS spectra of MoO_{2+x} after NRR test.

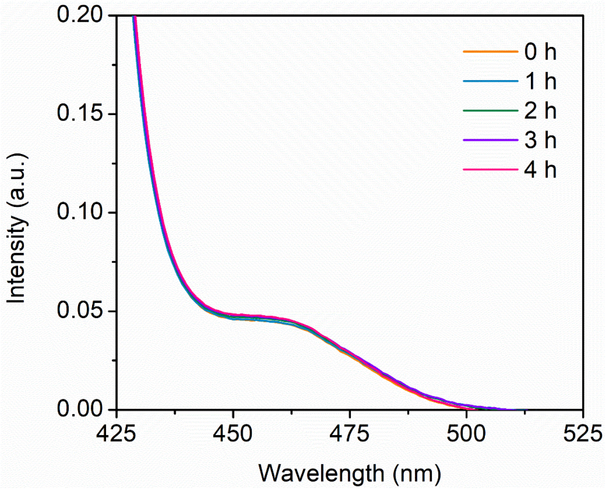

Figure S20. UV-vis spectra of the electrolytes for N_2H_4 after 0-5 h electrolysis.

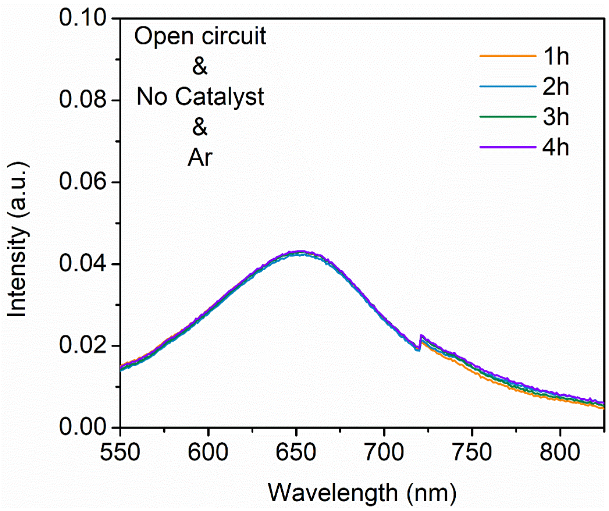

Figure S21. UV-vis absorption spectrum of indophenol blue colorimetry after a blank carbon cloth was electrolyzed without potential applied in Ar-saturated 0.1 M KOH electrolyte.

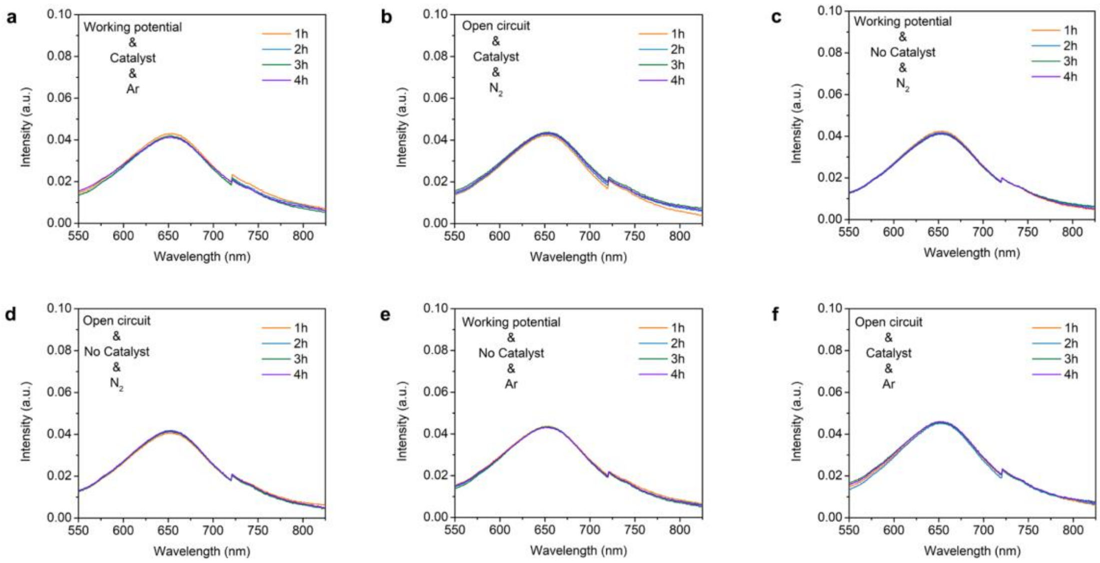

Figure S22. UV-vis absorption spectrum of indophenol blue colorimetry without part of the necessary conditions, including working potential, catalyst and N_2.

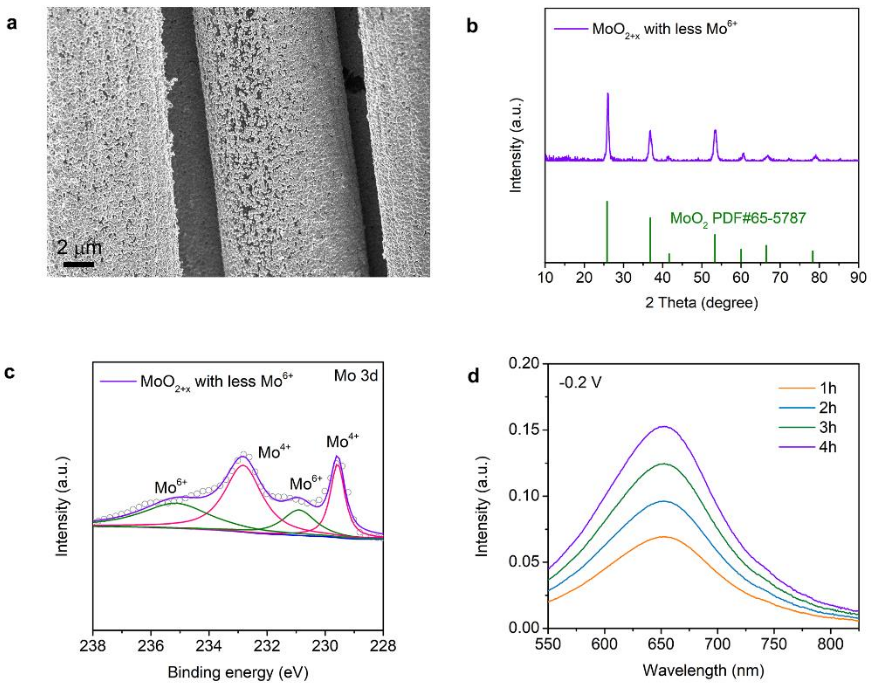

Figure S23. (a) SEM image of MoO2+x/CC (less Mo^{6+}). XRD pattern (b) and Mo 3d XPS spectra (c) of MoO_{2+x} (less Mo^{6+}). (d) UV−vis spectra of the electrolyte stained with indophenol indicator at different time durations at the potential of −0.2 V versus RHE by using MoO_{2+x} (less Mo^{6+}) as catalyst.

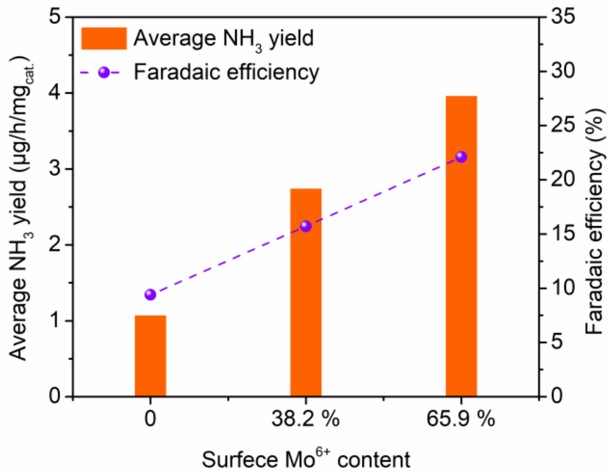

Figure S24. NH_3 yields and FEs of MoO_{2+x} with different Mo^{6+} content at the potential of -0.2 V vs. RHE.

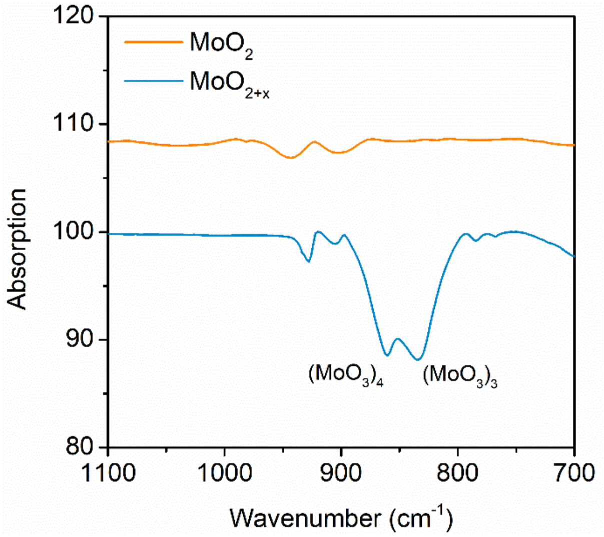

Figure S25. FT-IR spectrum of MoO_{2+x} and MoO_2.

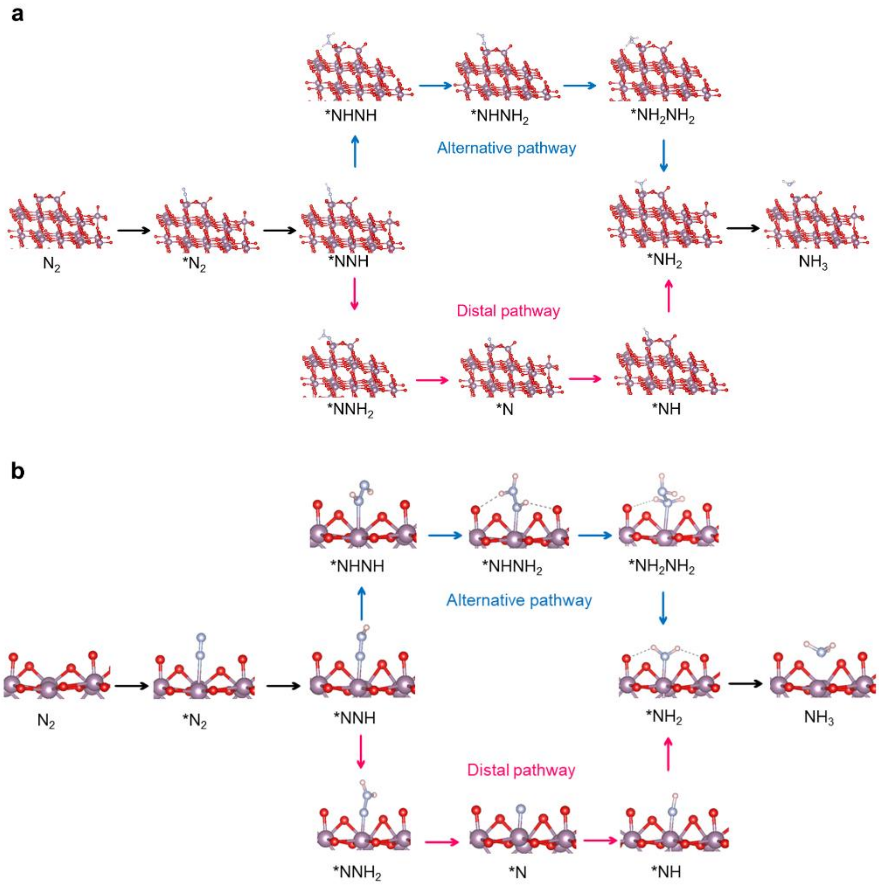

Figure S26. The two possible NRR pathways of alternative pathway and distal pathway on MoO_{2+x} (a) and MoO_2 (b). Purple, red, grey, and pink atoms represent Mo, O, N and H atoms, respectively.

Table S1. Ammonia yield rate and FEs of MoO_{2+x} and MoO_2 in the potential range from -0.1 to -0.5 V vs. RHE.

Catalyst	Potential (V vs. RHE)	Yield (μg/h/mg)	FE (%)
MoO_{2+x}	-0.1	0.49	10.6
	-0.2	3.95	22.1
	-0.3	2.11	9.7
	-0.4	1.31	4.2
	-0.5	1.02	0.8
MoO_2	-0.1	0.28	8.3
	-0.2	1.07	9.4
	-0.3	0.69	5.7
	-0.4	0.47	1.9
	-0.5	0.46	0.5

Table S2. Ammonia yield rate and FEs of MoO_{2+x} under different mass loading at -0.2 V vs. RHE.

Mass loading (mg/cm^2)	Yield (μg/h/mg)	FE (%)
0.1	0.63	3.7
0.3	1.94	6.9
0.7	3.15	18.5
0.9	3.95	22.1
1.3	3.73	17.3
